# Serine protease inhibitor dipetalogastin-like from *Galleria mellonella* is involved in insect immunity

**DOI:** 10.1038/s41598-025-08159-z

**Published:** 2025-07-06

**Authors:** Jakub Kordaczuk, Michał Sułek, Paweł Mak, Bożena Pawlikowska-Pawlęga, Iwona Wojda

**Affiliations:** 1 Department of Immunobiology, Institute of Biological Sciences, Maria Curie-Sklodowska University, Akademicka 19 Str., 20-033 Lublin, Poland; 2 Department of Analytical Biochemistry, Faculty of Biochemistry, Biophysics and Biotechnology, Jagiellonian University, Gronostajowa 7 Str., 30-387 Kraków, Poland; 3 Department of Functional Anatomy and Cytobiology, Institute of Biological Sciences, Maria Curie-Sklodowska University, Lublin, Poland; 4 Present Address: Department of Virology and Immunology, Institute of Biological Sciences, Maria Curie-Sklodowska University, Akademicka 19 Str., 20-033 Lublin, Poland

**Keywords:** *Galleria mellonella*, *Pseudomonas entomophila*, Antimicrobial proteins and peptides, Serine protease inhibitor, Immunology, Zoology, Entomology

## Abstract

A new protein with immune properties was found in *Galleria mellonella* hemolymph. The so-far putative serine protease inhibitor dipetalogastin-like (GmSPID) was found in one fraction obtained after separation of hemolymph by RP-HPLC. Its amount depended on the immune status of the insect: it significantly increased after oral (10^3 CFU) and intrahemocelic (10 and 50 CFU) infection with entomopathogenic bacteria *Pseudomonas entomophila*. This was accompanied by up-regulation of the respective gene in the fat body of infected larvae. GmSPID was purified to homogeneity and characterised as a protein with immune properties. Among the three proteases tested, i.e. trypsin, elastase, and thermolysin, the strongest inhibition was observed toward trypsin. No inhibition toward the metalloproteinase thermolysin was detected, confirming that GmSPID is an inhibitor of serine proteases. Additionally, GmSPID was shown to have antimicrobial properties. At the concentration of 7 µM and 15 µM, it acted against *Pseudomonas entomophila*, *Pseudomonas aeruginosa*, *Bacillus thuringiensis*, *Escherichia coli*, and *Candida albicans* but not against *Staphylococcus aureus*. Moreover, with the use of atomic force, scanning, and transmission electron microscopy techniques, we present the effect of the GmSPID protein on the surface properties, shape, and ultrastructure of *P. entomophila* cells. The protein caused modest perforation of the cellular membrane, contributing to loss of its integrity. The mode of the GmSPID protein action as an antimicrobial compound and its role in *G. mellonella* immunity are discussed.

**Supplementary Information:**

The online version contains supplementary material available at 10.1038/s41598-025-08159-z.

## Introduction

Most organisms rely only on innate immunity, which is older than adaptive and evolutionarily conserved immunity. One of the most effective elements of innate immunity is the production and activity of defence compounds, including antimicrobial proteins and peptides (AMPs)^1^. Antimicrobial peptides usually have a molecular mass between 2 and 10 kDa and exhibit defence properties. They can be produced by invertebrate and vertebrate organisms as part of innate defence against invading pathogens. Most antimicrobial peptides are cationic; therefore, they can bind easily to negatively-charged bacterial membranes. Another important feature of AMPs is their amphipathic properties, which are necessary for their electrostatic and hydrophobic interaction with microbial phospholipid membranes^2,3^. There are several mechanisms of AMPs action. For example, these peptides may lead to perforation of microbial membranes, leading to loss of homeostasis and death. Several models of AMPs interactions with the cellular membrane have been described in the literature, with the best-known carpet-like, barrel-stave, and toroidal models^4^. Another mode of action is crossing the lipid bilayer and disturbance in cellular processes, such as replication, transcription, protein synthesis, or protein folding. Immune proteins and peptides are of great interest to biotechnologists due to their wide applications. They show significant potential as novel therapeutic agents capable of acting against human pathogens, viruses, and cancer cells^5–9^. Another use of antimicrobial compounds consists in coating implants or food packaging to prevent the growth of microorganisms^10–12^.

Insects are a very rich source of antimicrobial molecules. In the greater wax moth *Galleria mellonella*, more than 20 AMPs have been identified. These include cecropins, gallerimycin, galiomycin, moricin-like peptides, galliosins, lysozymes, anionic peptides, proline-rich peptides, and heliocin-like peptide^13–20^. The appearance of antimicrobial peptides in the hemolymph of infected insects is an element of host-pathogen interactions. Microbial pathogen-associated molecular patterns are recognised by insect pattern-recognition receptors, inducing signal transduction and leading to AMPs synthesis. Antimicrobial peptides are under the control of Toll and Imd pathways, which are triggered in response to infection recognition^21,22^. It is worth mentioning that AMPs in the hemolymph of infected insects act synergistically to increase their effectiveness^23,24^. Even though insects do not possess an equally high level of specificity against different microbes, in contrast to vertebrates, it has been shown that infection with different microorganisms induces the synthesis of a different repertoire of antimicrobial compounds secreted to hemolymph^25^. In addition to immunity, some antimicrobial molecules possess other functions in insects. The examples include apolipophorin III (apoLp-III), which is also involved in lipid transport and infection recognition^26–28^and GmCP8 acting as an opsonin and protease inhibitor^15,29^.

Entomopathogens secrete compounds that digest insect anatomical barriers, such as the cuticle or gut, allowing intruders to colonise the entire body and suppress its immune response^30^. Among enzymes secreted by entomopathogens are proteases, such as those secreted by *Beauveria bassiana*,* Bacillus thuringiensis*, and *Pseudomonas entomophila*, which are perceived as virulence factors^31–33^. Their role is not only to create a “gate of infection” but also to degrade immune proteins, including antimicrobial peptides. Interestingly, degraded insect hemolymph proteins, called “protfrags” serve as inducers of immune response, including the expression of the gene encoding IMPI (inducible metalloproteinase inhibitor)^34^. Its synthesis proves the multilevel nature of insect host-pathogen interactions^35^.

Among pathogens that may infect *G. mellonella*, there are Gram-positive and Gram-negative bacteria, including *B. thuringiensis* and *P. entomophila*, respectively, both infecting larvae orally, and *B. bassiana* and *Metharizium anisopliae* fungi able to infect through the cuticle. During their antagonistic co-evolution with *G. mellonella*, both the pathogens and the host have developed tools and mechanisms for enhanced virulence and defence, respectively.

In search of new microbicidal compounds, we infected *G. mellonella* larvae with entomopathogenic bacteria *P. entomophila*. The infection was done orally with two doses of 10^3^ and 10^5^ cells/larvae and by intrahemocelic injection of 10 and 50 *P. entomophila* cells. In both cases, the hemolymph was analysed for the presence of induced compounds 24 h after the infection. Here, we present identification and characterisation of the *G. mellonella* serine protease inhibitor dipetalogastin-like (GmSPID) and describe its defence properties as an antimicrobial agent and an inhibitor of serine proteases for the first time.

## Results

### The level of the GmSPID protein in *G. mellonella* hemolymph depends on the immune status of the insect

**Fig. 1:**
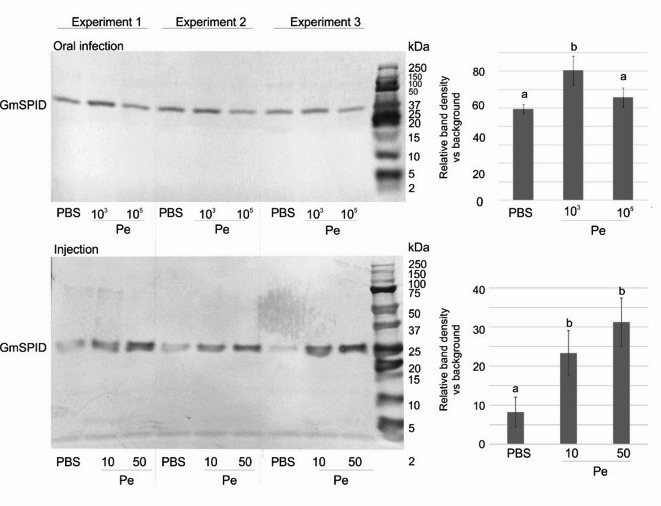
Comparative quantitative analysis of fraction No 25 from RP-HPLC separations after electrophoretic separation followed by transfer of separated proteins on the PVDF membrane. Left: stained images of membranes, right: quantitative analysis of GmSPID. The control samples (PBS administration), samples from oral infection with 10^3^ and 10^5^ of *P. entomophila*, and injection with 10 and 50 cells of *P. entomophila* are shown from three experiments. Mean values ± SD are shown; different letters show statistical difference (*p* < 0.05, ANOVA, Tukey’s test; *n* = 3). Uncropped and uncut images are presented in Supplementary Fig. S3.

During the comparison of reversed-phase high pressure liquid chromatography separations (RP-HPLC) of hemolymph from infected and non-infected *G. mellonella* larvae, we observed that the level of proteins in fraction No 25 was repeatedly higher in individuals infected with 10^3^ CFU (colony forming units) of *P. entomophila* orally via force feeding and via intrahemocelic injection (10 and 50 CFU, Supplementary Figs. S1 and S2). The fraction contained a protein with molecular weight of ca. 25 kDa and received the working name p25 as a protein from fraction No 25^36^. Its amount depended on the immune status of the insect (Fig. 1 and Supplementary Fig. S3). The level of the protein in the orally infected animals increased significantly after the infection with 10^3^ CFU and was at the control level after the application of the higher dose (10^5^ CFU). In turn, its level increased after the infection with both doses *via* injection.

The protein was cut out from the membrane and subjected to mass spectrometry (MS) and to N-terminal sequencing by Edman degradation. The obtained result allowed us to identify the protein as a putative serine protease inhibitor dipetalogastin-like (GmSPID) with predicted molecular weight of 23.8 kDa (Supplementary Fig. S4). Its similarity to other proteins is presented in Supplementary Table.

**Fig. 2:**
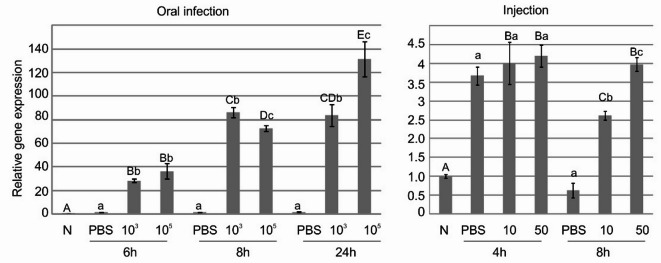
Relative expression of a gene encoding GmSPID in the fat bodies of *G. mellonella* larvae after oral infection with 10^3^ and 10^5^ CFU (left) and intrahemocelic injection of 10 and 50 cells (right) of *P. entomophila*. Mean values ± SD from three assays are shown. Significant differences are indicated by different letters (*p* < 0.05 One-Way ANOVA, Newman–Keuls post-hoc tests; *n* = 3); lower case letters show significance between different groups at the same time point, upper-case letters show significance between different time points in infected larvae, taking into account naive larvae.

Further, we checked whether the increased protein level in the infected larvae was correlated with its gene expression in fat bodies—the primary organ of insects responsible for the synthesis of effector molecules during systemic immune response. We found a 30–130-fold increase in the amount of transcripts 6 and 24 h after the force feeding with 10^3^ and 10^5^ cells of *P. entomophila*, in comparison to the PBS-fed and naive larvae. At the 24 h time-point, the relative transcript level was dose dependent. On the other hand, the injection of 10 and 50 cells to *G. mellonella* hemolymph resulted in a 2.5- and 4-fold increase in the amount of transcripts 8 h after the injection. Earlier, i.e. 4 h after the injection, the level also increased but to the same extent as after the injection of PBS buffer in which the bacteria were resolved (Fig. 2).

### Purification and biological activity of GmSPID

**Fig. 3:**
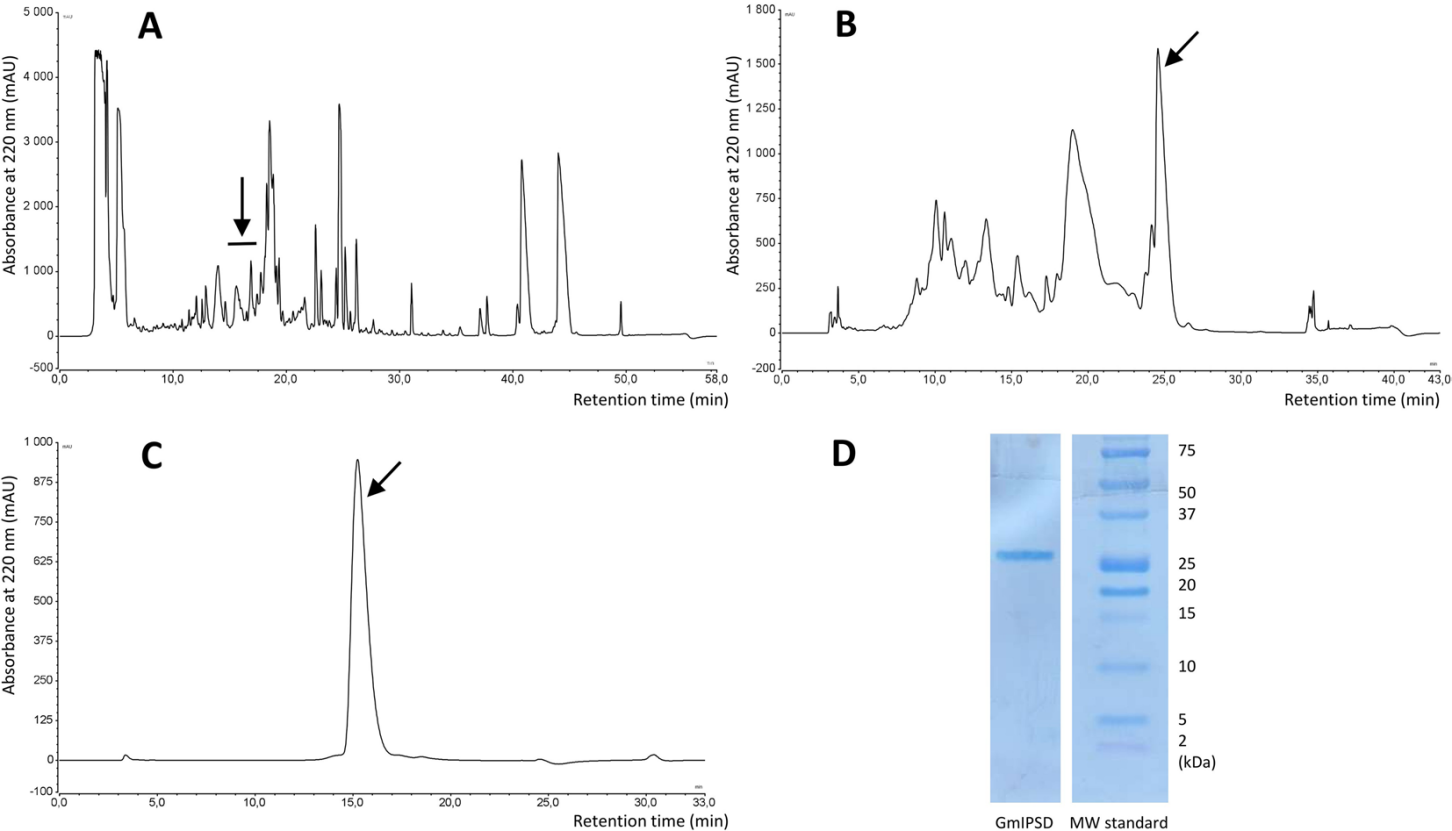
Purification of the GmSPID protein by RP-HPLC chromatography. (**A**–**C**) Chromatograms from three consecutive steps of purification; the collected fractions are denoted by arrows; (**D**) SDS-PAGE electrophoretic resolution of purified protein after transfer to the PVDF membrane. The details of the procedure are described in the Materials and methods section. A cut fragment containing purified GmSPID protein aligned with molecular standards is shown. An uncropped and uncut membrane is presented in Supplementary Fig. S5.

GmSPID was purified from the hemolymph of *G. mellonella* larvae in three chromatography steps presented in Fig. 3A–C. The obtained preparation was homogenous in SDS-PAGE, which showed a single band localised slightly above the 25 kDa marker protein (Fig. 3D and Supplementary Fig. S5). The N-terminus of the protein was sequenced by Edman degradation and confirmed that the purified protein was GmSPID.

**Fig. 4:**
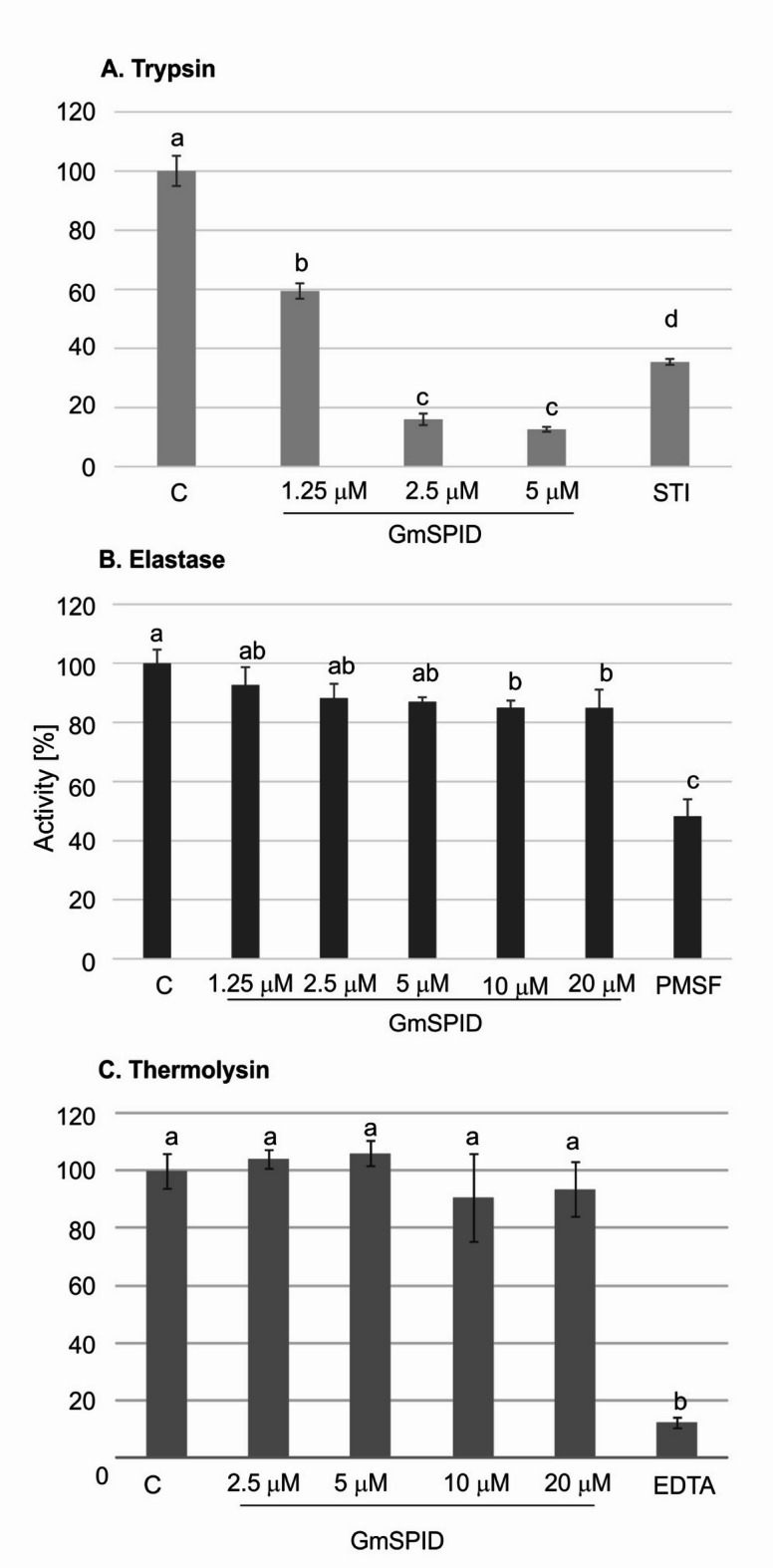
Effect of different concentrations of GmSPID on the activity of trypsin (**A**), elastase (**B**), and thermolysin (**C**). The assay was performed as described in the Materials and methods. 1 µM STI (**A**), 1 µM PMSF (**B**), and 500 µM EDTA (**C**) were used as positive controls of enzyme inhibition. The control group (100% relative activity) contained water instead of GmSPID. Different letters show statistically significant results (*p* < 0.05, ANOVA, post-hoc Tukey test; *n* = 3).

Since GmSPID is a putative inhibitor of proteases, we checked its activity toward serine proteases (trypsin and elastase) and a metalloproteinase (thermolysin). The protein at the concentration of 1.25 µM inhibited the activity of trypsin to 60% of its control value. An increase in the concentration of GmSPID to 2.5 µM and 5 µM resulted in a decrease in enzymatic activity to about 17% and 12%, respectively. Only slight inhibitory activity (about 85% of the control) was detected toward elastase at the concentration of 5 µM GmSPID, but the increase in its concentration to 20 µM did not result in further inhibition of proteolytic activity. No inhibition of thermolysin was observed at the GmSPID concentration used (Fig. 4).

**Fig. 5:**
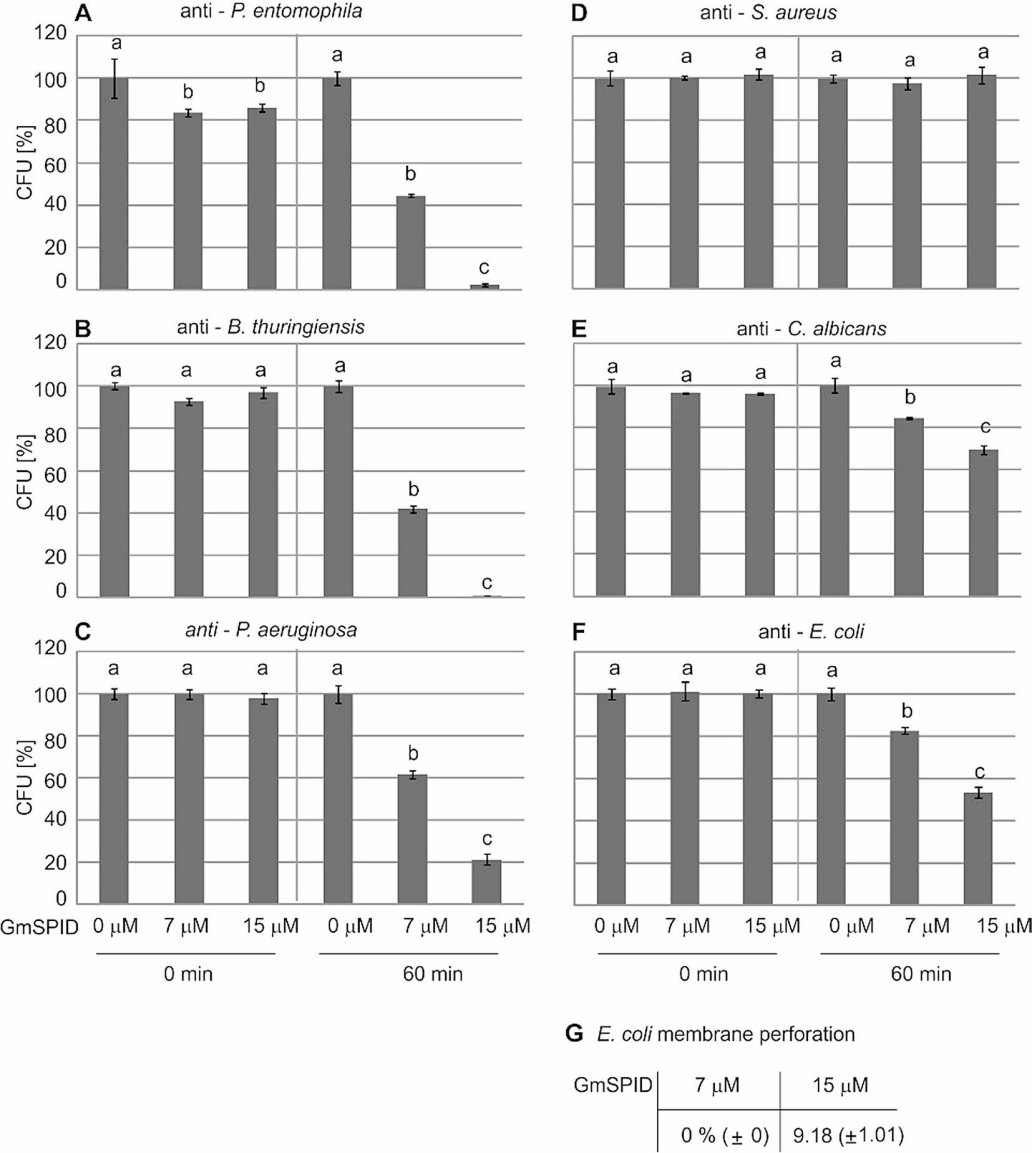
Antimicrobial activity of GmSPID (**A**–**F**) and bacterial membrane permeabilisation determined by measuring β-galactosidase leakage into the medium after treatment with GmSPID (**G**). The purified protein was incubated with a given microorganism as described in the Materials and methods section. The control suspension (Time 0) and the suspension after 60-min incubation (Time 60) were plated, and the numbers of grown CFU were counted. The results are shown in relation to the number of CFU grown after incubation with water instead of the peptide at the respective time-points. The results are shown as a percentage of CFU grown after the incubation with the GmSPID protein in relation to the average number of CFU without the addition of the peptide. Mean values ± SD are shown. Different letters show statistical differences (*p* < 0.05, ANOVA, Tukey’s test; *n* = 3). The level of perforation of dead bacteria was assumed as 100%. The values represent means from three permeabilisation assays ± SD.

Further, we found that GmSPID also exhibited antimicrobial potential against various microorganisms (Fig. 5A–F). The incubation of *P. entomophila* with GmSPID at the concentration of 7 µM reduced the number of CFU to about 40% of the number observed in the control group. The use of a higher concentration of GmSPID (15 µM) almost completely limited the bacterial growth in comparison to the control cells incubated without the protein (Fig. 5A). Similar activity was found toward the other entomopathogenic bacteria *B. thuringiensis* (Fig. 5B). GmSPID also showed activity against the human opportunistic pathogen *Pseudomonas aeruginosa*, reducing the number of CFU to 60% and 20% at the 7 µM and 15 µM concentrations, respectively (Fig. 5C) and toward *Escherichia coli*, reducing the number of CFU to 80% and 50%, respectively (Fig. 5F). The weakest activity was observed toward yeast-like fungi *Candida albicans*, where the number of CFU was reduced to 85% and 70% at 7 µM and 15 µM of GmSPID, respectively (Fig. 5E). The investigated protein showed no activity toward *Staphylococcus aureus* at the concentration tested (Fig. 5D).

The *E. coli* strain JM83 used for testing the antibacterial activity of GmSPID carries plasmid pCH110 encoding ß-galactosidase. No ß-galactosidase activity was detected in the presence of 7 µM GmSPID, while the activity of ß-galactosidase was detected at the higher concentration, i.e. 15 µM, which reflects the perforation of *E. coli* membranes. The perforation was estimated as above 9% in relation to that induced by the presence of cecropin B (Fig. 5G).

### GmSPID-induced changes in the structure of *P. entomophila* cells

**Fig. 6:**
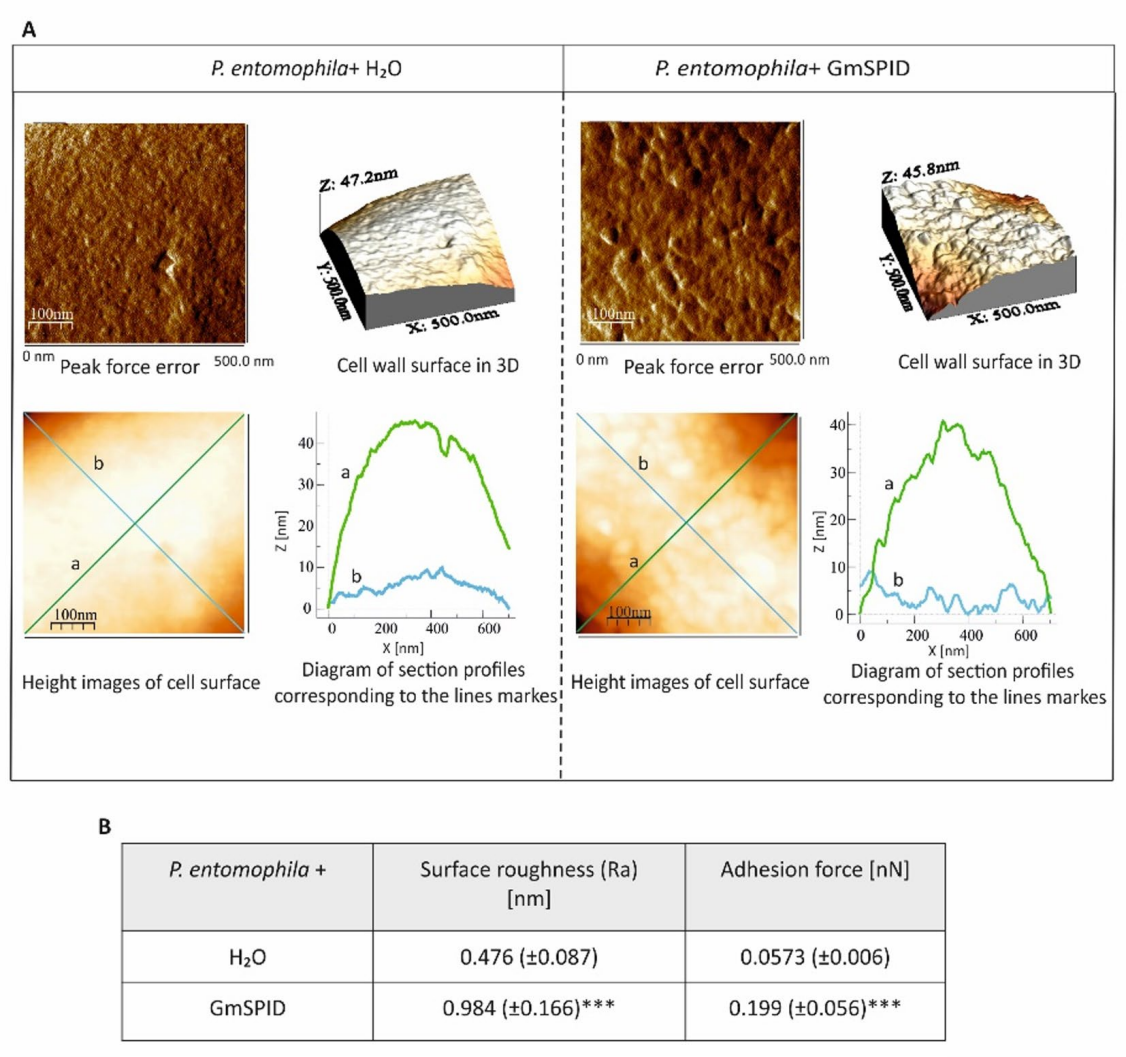
Effect of the GmSPID protein on *P. entomophila* cell surface topography. *P. entomophila* in the logarithmic growth phase was incubated with water (control) or with GmSPID for 60 min. Then, the cells were imaged by AFM. The peak-force-error, three-dimensional, and height images are presented (area of 500 nm×500 nm) as well as cell surface change profiles measured along lines “a” and “b” marked in the height image. The table shows biophysical parameters of the cell surface, such as roughness and adhesion force values. Mean values ± SD are shown, *n* = 60. Significant differences are indicated at *p < 0.05, **p < 0.01, and ***p < 0.001 (Mann–Whitney U test). The images were taken using Nanoscope Analysis vl. 40 (Veeco); the section profiles and three-dimensional (3D) images of the cells were generated using WSxW 5.0 sofware (Nanotec, Spain).

Using atomic force microscopy (AFM), we analysed changes in the morphology and biophysical parameters of the *P. entomophil*a cell surface after the incubation with GmSPID (Fig. 6). The peak force error images show differences in the structure; after the treatment with GmSPID, the bacterial surface lost its regular structure, and a number of bulges in the form of lumps were visible. Since the distance from the lowest to the highest peak was similar, as evidenced by the values on the Z axis (see 3D structure), it can be assumed that the action of the analysed protein resulted in cell shrinking and “wrinkling” of the cell surface. The surface texture measurements along lines “a” and “b” shown in the height image of the surface presented graphically confirmed these observations. The lines were definitely more serrated, which indicates large unevenness of the surface of the bacterial cells (Fig. 6A). Additionally, the topography alterations were accompanied by changes in biophysical parameters, such as bacterial surface roughness, which increased twice, and changes in the strength of the needle adhesion to the bacterial surface, which increased approximately three times in the GmSPID-treated *P. entomophila* cells (Fig. 6B).

**Fig. 7:**
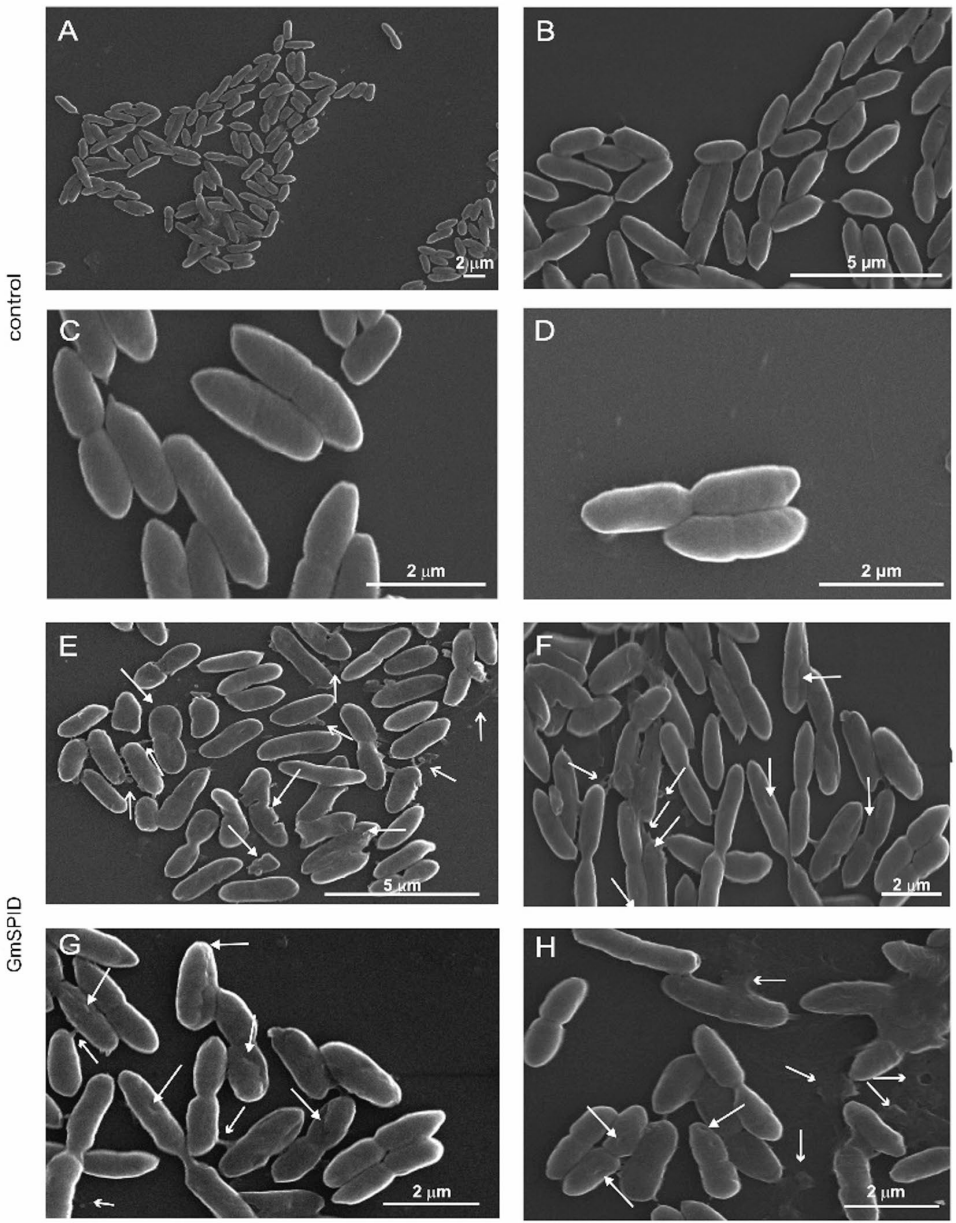
Effect of GmSPID on *P. entomophila* morphology. Control cells (**A**–**D**) and cells treated with 7 µM GmSPID (**E**–**H**). (**A**) many control cells showing a well-maintained cell surface; (**B**–**D**) enlarged view of control cells showing a smooth and intact cell surface; (**E**,**F**) many cells treated with GmSPID showing different changes in morphology; (**G**,**H**) enlarged view of bacteria presenting changes visible on the surface of the microorganisms after the treatment with GmSPID. Deformed cells with a corrugated surface, indentations, and protrusions are indicated by arrows with filled arrowheads. Some substances in the immediate vicinity of the cells are indicated by open arrowheads.

To check whether the changes in the topography and properties of the cellular envelope observed in AFM resulted in changes in cellular morphology, we employed scanning (SEM) and transmission electron microscopy (TEM). The analysis of the SEM images indicated that the cells from the control samples displayed a smooth and intact surface (Fig. 7A–D). They maintained their typical quite narrow and elongated shape. After the incubation with the protein, cells with disturbed morphology were visible. The surface of the cells had ridges and/or grooves. There were also some small or bigger indentations and pits in the cells. Along with the smaller or bigger depressions on the surface, protrusions with different appearance were observed in some cells. Some substance was clearly visible in the immediate vicinity of the cells (Fig. 7E–H).

**Fig. 8:**
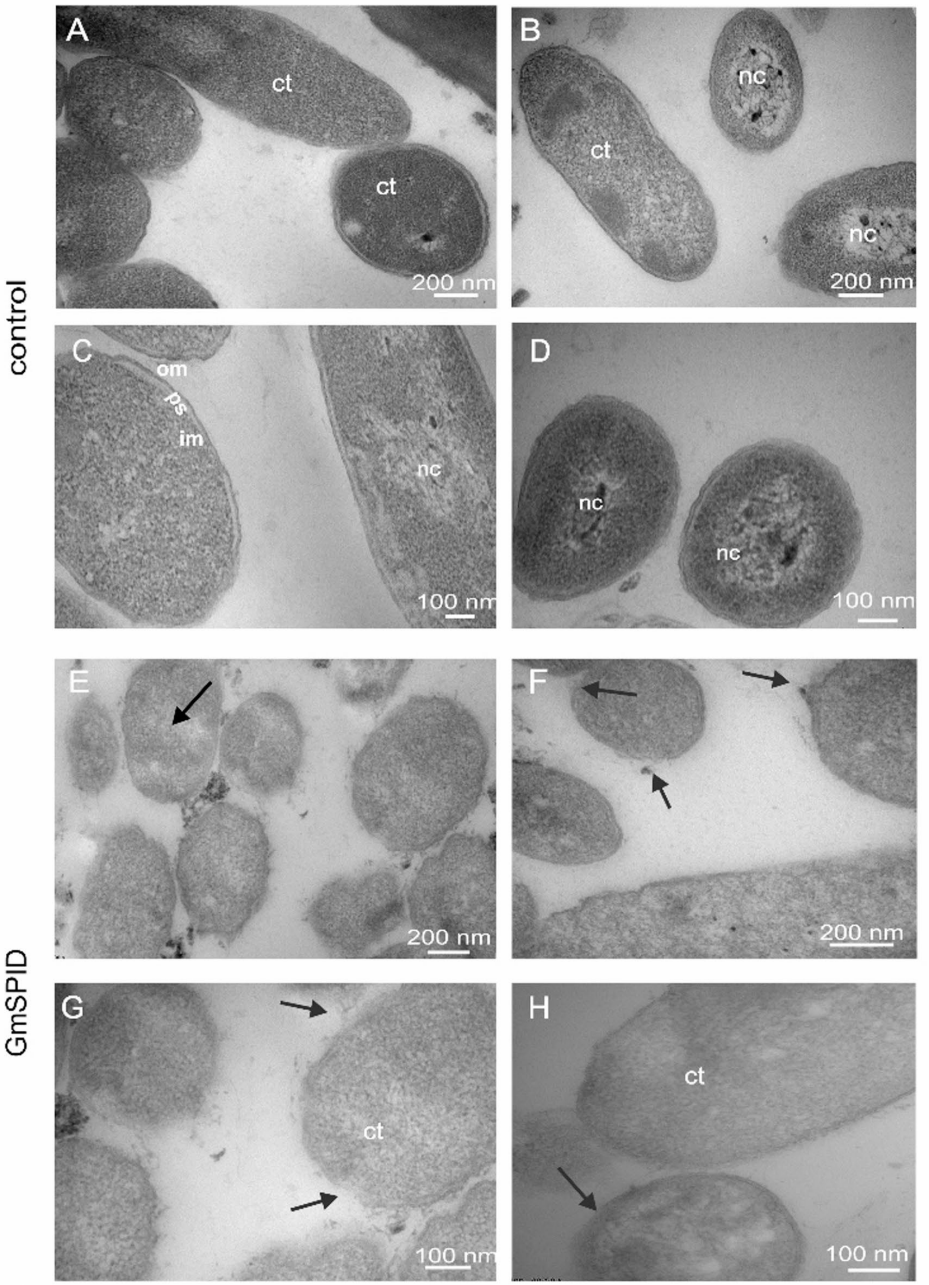
Influence of the GmSPID treatment on *P. entomophila* cells—TEM images. (**A**–**D**) show images of untreated (control) cells with an intact cell envelope: outer and inner membrane (om, im—respectively), periplasmic space (ps), granular cytoplasm (ct), and clearly distinguishable nucleoids (nc). The cells retain their structural integrity. Electron micrographs (**E**–**H**) show cells upon treatment with 7 µM GmSPID. The cell envelope is apparently disturbed (arrows) and the cytoplasm exhibits lower electron density. The changes are indicated by arrows.

The observations of the *P. entomophila* bacterial cells under the transmission electron microscope (TEM) revealed that the control cells exhibited dense, granular cytoplasm. Additionally, these cells were surrounded by an intact cell envelope consisting of the outer cell membrane, the periplasm, and the inner plasma membrane. Within the cells, distinctive nucleoids were visible, and the cells retained their shape (Fig. 8A–D). After the 60-min incubation with the examined protein, some alterations of the ultrastructure were found. Uneven and less electron-dense cytoplasm was a characteristic feature of the examined cells. In the majority of the cells, there were some disruptions in the cell envelope accompanied by discontinuities. Corrugation of the surface layers was noticed in some cells (Fig. 8E–H).

Summarising, the GmSPID action resulted in significant changes in the *P. entomophila* structure, such as changes in the topography and physical parameters of the cellular surface, alterations in the cellular shape, and disruptions in its ultrastructure, including cytoplasm leaking.

## Discussion

Mutual interactions between pathogens and their hosts are determined by antagonistic co-evolution. While pathogens enhance their virulence factors, hosts that are repeatedly infected improve their defence mechanisms^37,38^. The hemolymph of infected insects is therefore a valuable source of bioactive compounds^1^.

We report here the identification and characterisation of the so far putative protein, XP_026756133.1, from the hemolymph of immune-challenged *G. mellonella* larvae. According to the NCBI blastp service, the putative protein is composed of 219 amino acids, contains four Kazal domains, and is named Kazal-type serine protease inhibitor dipetalogastin-like (GmSPID, see Supplementary Fig. S6). We found that the level of GmSPID in the larval hemolymph was significantly higher in larvae that were orally infected with 10^3^ but not 10^5^ CFU of *P. entomophila* and after the injection of 10 and 50 CFU of these bacteria. The analysis of the expression of the gene encoding GmSPID, namely LOC113516003 in the fat bodies, revealed the highest induction after the oral infection; it increased 30–130 times after the 10^3^ and 10^5^ ingested doses, respectively. Its amount changed only 2.5 to 4 times after the intrahemocelic injection with 10 and 50 cells, respectively. The difference in the induction level observed for the two infection routes was probably determined by the difference in the dose of the microorganism administered. Oral infection requires a higher dose than injection to have a similar impact on insect physiology. Previously, we demonstrated the courses of survival curves of *G. mellonella* depending on the dose and route of infection^39^. Moreover, the oral infection resulted in a time-dependent increase in the amount of transcripts. The GmSPID gene expression increased; however, 6 and 8 h post-infection, it remained at a similar level regardless of the infection dose, while 24 h post-infection, the relative amount of transcripts was higher after the administration of the higher dose. This suggests that the infection-induced immune response of *G. mellonella* is at a similar (already maximum? ) initial level but could be maintained longer when the initial dose is higher. The other possibility could be the difference in the stability of transcripts, which may take a longer time in larvae that ingested 10^5^ cells than 10^3^ cells. In our opinion, proliferating bacteria or compounds secreted by microorganisms are more likely to contribute to the prolongation of the time of increased gene expression than to the protection of its transcript from degradation. The next conclusion is that, despite the high gene expression at 24 h after the oral application of the 10^5^ CFU dose, the amount of protein is at the control level. This may indicate the action of pathogen’s virulence factors, i.e. proteases, as we reported previously for other hemolymph proteins in naturally infected larvae^39,40^. Their activity is more pronounced when the higher dose is ingested.

The approximately four-fold induction was observed 4 h after the injection of larvae with both PBS and bacteria. It is likely that the product of the LOC113516003 gene, i.e. GmSPID, regulates wound healing, which is more likely in the light of the fact that the protein is a protease inhibitor. At 8 h after the injection, we observed dose-dependent gene expression, as we did 24 h after the ingestion of the bacteria. In the case of injection, we chose earlier time-points, since systemic immune response is induced earlier when microorganisms do not have to penetrate the intestine barrier^30,41^.

The purified GmSPID had molecular weight of about 25 kDa, which is consistent with predicted molecular weight of XP_026756133.1 protein, i.e. 23.7 kDa. First, we checked the ability of the purified protein to inhibit certain proteolytic enzymes. Out of the three proteases tested, the strongest inhibition was observed toward trypsin, weaker inhibition was noted toward elastase (both serine proteases), and no inhibitory effect was found toward the zinc metalloproteinase thermolysin.

Serine proteases contain serine that acts as a nucleophilic residue in the active site of the enzyme^42^. These enzymes generally have extracellular functions, and are secreted outside cells. In insects, they are involved in a variety of functions, including digestion, metamorphosis, and immunity. Consequently, serine protease inhibitors (SPIs) co-operate in the regulation of these processes. A number of SPIs have been reported in insect hemolymph, the midgut and the silk glands^43–45^.

Insect SPIs are classified into several families: Kunitz-type, Kazal-type, serpin-type, and pacifastin-related^46–49^.

Kazal-type serine protease inhibitors (KPIs), including dipetalogastin from *Dipetalogaster maximus*, are grouped into the I1 MEROPS family and named as in a study^50^ describing pancreatic secretory trypsin inhibitor SPINK1. They contain one or more Kazal domains linked together by peptide spacers of variable length. Each Kazal domain shares six conserved cysteine residues forming three intra-domain disulphide bridges and acts as a substrate analogue that binds competitively through its reactive site loops to the active site of the cognate proteinase, forming a very stable complex^51^. It is worth noting that some Kazal domains are inactive towards the proteinases tested. For example, the Kazal domain 1 of SPIPm2 from *Penaeus monodon* did not inhibit protease activity^,^ while other domains were differently active against specific proteases. Moreover, domains 2, 4, and 5 contributed to the bacteriostatic properties of this SPIPm2^52^. This may be similar to our GmSPID inhibitor, which effectively inhibited trypsin, but elastase was inhibited only to a certain extent. However, we cannot exclude the possibility that the slight inhibition detected in the study was due to the contamination of the elastase sample with trypsin, as mentioned by the producer in the product description.

Serine proteases take part in at least three aspects of insect immune response. They are necessary for the cleavage of the cytokine Spatzle for Toll pathway activation as well as the cleavage of prophenoloxidase and its transformation into active phenoloxidase - an enzyme necessary for melanin synthesis. Finally, they take part in the hemolymph coagulation process^53^. It is likely that this immune-regulated protein is synthesised to down-regulate some of these processes. Coagulation and melanin synthesis are also part of wound healing, which correlates with the increase level of GmSPID transcripts after aseptic injury^54,55^. Another possibility is that GmSPID acts on some serine proteases of pathogen origin. Proteases, lipases, and chitinases are produced by entomopathogens as virulence factors^30,33^. Their role is to break the host’s anatomical barriers and to destroy its immune-related proteins and peptides. In *P. entomophila*, many virulence factors are under the control of the global regulatory system (Gac) and the *Pseudomonas* virulence factor (Pvf) gene cluster^56^. It has been shown that some proteases are under the control of the latter cluster. These include alkaline metalloprotease AprA encoded by the *PSEEN 1550* gene, i.e. an enzyme cleaving the virulence factor monalysin destroying the gut of infected insects. The other protease under the control of the Pvf cluster is serine protease PspB encoded by the *PSEEN 3027* gene. Its expression was found to increase more than 2-fold in the delta *prf A-D* strain^57^. The *PSEEN 3027* gene has been classified as encoding a protein involved in *P. entomophila-Drosophila melanogaster* interaction during the infection process^58^. Other serine proteases genes classified by the same authors include *PSEEN 3028* and *PSEEN 3027*, encoding putative autotransporters^57^ and *PSEEN 4433* encoding a subtilisin-like serine protease. The exact roles of these secreted proteases in the infection process are unknown, but it is very likely that the inhibition of serine protease(s) by GmSPID contributes to reduced virulence of *P. entomophila* and is a part of the insect immune response. This possibility is supported by the fact that GmSPID also possesses antimicrobial activity. The strongest activity was observed toward natural pathogens of the greater wax moth, i.e. *B. thuringiensis* and *P. entomophila*. At the concentration of 7 µM, it reduced the number of CFU to 40% in relation to cels incubated with water, while barely any colonies were observed after the incubation with GmSPID at 15 µM. Its activity was also observed toward human opportunistic pathogens, such as *P. aeruginosa*, *C. albicans*, and *E. coli*, but not toward *S. aureus*.

According to their secondary structure, antimicrobial compounds can be divided into four groups: α-helical ones, β-sheet peptides, compounds with αβ-structure, and linear extended molecules rich in one or more amino acids^59–61^. Simplifying, α-helical peptides damage microbial membranes through carpet, barrel-stave, or toroidal models^62^. The β-sheet antimicrobials, whose structure is stabilised by disulphide bridges, act in a variety of ways, including binding to membrane lipids and prevention of cell-wall formation. They may translocate along lipid bilayers causing formation of temporary pores^63,64^. The linear extended peptides rather do not interact with the pathogen’s membrane but permeate membranes and interact with cytoplasmic proteins^46–49^.

Taking into account the putative structure of GmSPID (see Supplementary Fig. S6), which consists of β-sheets and the presence of Kazal domains, we can initially classify GmSPID to the second group of antimicrobials mentioned above. This group includes *inter alia* defensins, the well-known antimicrobial peptides present in invertebrates and vertebrates. Among the cysteine-rich antimicrobials found in *G. mellonella*, there are gallerimycin, galiomicin, and defensin-like peptides^1^. The formation of temporary pores by GmSPID corresponds with our findings of only 9% of membrane permeabilisation in comparison to cecropin B. Cecropins disrupts microbial membranes via the carpet-like mechanism^65^which results in significant loss of intracellular fluids. Rather weak leakage of cellular fluids in comparison to antimicrobial activity suggests formation of rather temporary pores, as mentioned above, or their low number.

Based on the AFM analysis, GmSPID causes significant changes in the topography of the bacterial surface, resulting in altered morphology. After the GmSPID treatment, the *P. entomophila* surface became irregular and rougher. Its roughness increased about twice and the adhesion of the probe to the bacteria increased four times, indicating alterations in the nanomechanical properties of the cellular envelope. As reported before, the antimicrobial peptide cecropin D from *G. mellonella*, changes the topography and nanomechanical properties of the cellular surface upon binding to *E. coli* JM83 in a dose- and time-dependent manner^66^. Changes in *E. coli* surface properties were also observed after treatment with cecropin A and lysozyme from *G. mellonella*^23,67^. Moreover, apolipophorin-III (apoLp-III), a multifunctional protein with direct antimicrobial properties, increased the roughness of the cell surface in several microbial species^27,28^. AFM also revealed altered cellular topology of *P. entomophila*, *B. thuringiensis*, and *C. albicans* exposed to GmCP8 and the first two bacteria exposed to Kazal peptide Pr13a^15,20^. The GmSPID-induced alteration in the cellular envelope resulted in a change in cellular morphology, as revealed by SEM. A liquid was usually visible near cells with morphology that was unchanged at a first glance. It is likely that the observed changes revealed subsequent stages of the GmSPID action. First, the perturbation of the cellular envelope occurs, causing the contents of cells to leak out. This in turn causes loss of turgor affecting the cellular shape, as observed in SEM. Indeed, by using the *E. coli* JM83 carrying plasmid pCH110 encoding β-galactosidase, we detected membrane perforation. As a result, disruption of the ultrastructure of *P. entomophila* cells was visible in TEM, showing the loss of bacterial cell integrity. The GmSPID-treated cells had less electron-dense cytoplasm and exhibited disruption and discontinuities in cellular membranes. In most cells, the nucleoid was not as clearly visible as in the control cells.

In the light of the presented results, the question arises whether the antimicrobial activity of GmSPID is directly correlated with its inhibitory activity toward certain proteases, e.g. whether it acts via inhibition of certain microbial protease(s), causing biochemical perturbation resulting in disturbed cellular morphology and death. The other possibility is that the structure of GmSPID, including Kazal-domains stabilized by disulphide bridges, confers antimicrobial activity to the protein independently of its protease inhibitory properties.

Certainly, further studies are required to unravel the mechanism of the antimicrobial action of this immune-relevant protein.

## Materials and methods

### Insects, microorganisms, and infection

The greater wax moth *Galleria mellonella* (Lepidoptera: Pyralidae) was maintained in a continuous laboratory culture. The larvae were reared on honeybee nest debris at 28 °C in darkness. For infection, an overnight culture of *P. entomophila* (Table 1) was sedimented (8500 ×g), washed with Phosphate Buffered Saline (PBS; 140 mM NaCl, 2.68 mM KCl, 10 mM Na2HPO4, 1.76 mM KH2PO4 in pH 7.4), and suspended in PBS to the density of 10^3^ and 10^5^ colony forming units in 10 µl for oral infection by force feeding or 10 and 50 cells in 5 µl for injection. The number of cells was estimated according to optical density (OD) at 600 nm for oral infection and with a cell counter Muse Cell Analyzer (MERCK Millipore) for intrahemocelic injection, followed by plating the cells and calculation of CFU. Infection by force feeding was done under the stereoscopic microscope by gently introducing the needle of a Hamilton syringe in the larval month opening. For the intrahemocelic route of infection, the larvae were injected with the use of a Hamilton syringe in the last or last-but-one proleg previously sterilised with 70% ethanol.

For immunisation with non-pathogenic microorganisms, *E. coli* (CGSC5165) and *Micrococcus luteus* (see Table 1) were grown overnight and then sedimented as described above. A combined pellet containing both microorganisms was used for immunisation by pricking the larvae in the last-but-one proleg with a needle dipped into the pellet.

The infected larvae were placed on a sterile filter paper in well-ventilated plastic boxes with access to food and kept at 28 °C for 24 h. Larvae which regurgitated the ingested suspension or were bleeding extensively after the injection or pricking were removed from the experiment. As a control, a respective volume of PBS was force fed (10 µl) or injected into the larval hemocel (5 µl). The microorganisms used in this study are summarised in Table 1.

**Table 1:** Microorganisms used in this study.

Microorganism	Source/description	Growth conditions	Use in this study
*Pseudomonas entomophila L48*	Frederic Boccard, CNRS, France ^68^	LB, 30 °C	Oral and intrahemocelic infection of *G. mellonella*; tests of antibacterial properties of GmSPID
*Escherichia coli D31*	CGSC5165, Genetic Stock Centre, New Haven, CT, USA	LB, 37 °C	Immunisation by pricking for preparative purification of GmSPID
*Micrococcus luteus*	ATCC 4698	LB, 37 °C	Immunisation by pricking for preparative purification of GmSPID
*Bacillus thuringiensis*	Subsp. kurstaki HD1, Bacillus Genetic Stock Centre, The Ohio State University, Department of Biochemistry	LB, 37 °C	Tests of antibacterial properties of GmSPID
*Candida albicans*	ATCC 10231	YPD, 37 °C	Tests of antifungal properties of GmSPID
*Escherichia coli JM83* carrying plasmid pCH110	Pharmacia-Amersham	LB, 37 °C with ampicillin	Tests of antibacterial properties of GmSPID
*Pseudomonas aeruginosa*	ATCC 27853	LB, 37 °C	Tests of antibacterial properties of GmSPID
*Staphylococcus aureus*	Collection of the Department of Genetics and Microbiology, UMCS, Lublin, Poland	LB, 37 °C	Tests of antibacterial properties of GmSPID

### Collection of hemolymph and dissection of the fat body

For hemolymph collection, the larvae were anesthetised by cooling down at 4 °C, and their surface was wiped with 70% ethanol. Twenty microlitres of hemolymph from each larva were pooled in one Eppendorf tube. The Eppendorf tubes contained a few crystals of phenylthiourea to prevent hemolymph melanisation. Then, the hemolymph was centrifuged at 200×g at 4 °C to pellet hemocytes, and again at 20,000×g for 10 min to remove all debris. The hemolymph prepared in this way was further used for the extraction of low molecular weight components and comparative RP-HPLC.

The fat body was isolated from anesthetised larvae under ice-cold sterile Ringer’s solution (172 mM KCl, 68 mM NaCl, 5 mM NaHCO3, pH 6.1, osmolarity 420 mOsm). Organs from five larvae in each group (in every experiment) were pooled in ice-cold Ringer’s solution in Eppendorf tubes. Then, the liquid was removed and the organs were quickly frozen in liquid nitrogen for at least 10 min and stored at – 80 °C until use.

### Extraction of low molecular weight hemolymph components for Tris-Tricine electrophoresis

Extraction was done as previously^20,39^. Shortly, insect hemolymph was diluted ten times in a mixture containing methanol, water, and acetic acid (90:9:1 v/v). After sedimentation of high molecular weight proteins (20,000×g for 30 min, 4 °C), the upper fraction was lyophilised in a BETA 1–8 LD plus freeze dryer (Christ, Germany). Further, to remove lipids, the dry pellet was placed in 0.1% (vv) trifluoroacetic acid (TFA) as described before^20,39^. An equal volume of n-hexane was added, and the samples were mixed and centrifuged for 15 min at 4 °C. The lipids present in the upper fraction were removed, ethyl acetate was added to the bottom part, and the samples were mixed and centrifuged again. The lipid-free bottom part was then freeze-dried. After separation of protein by RP HPLC performed as described previously^15^Tris-Tricine electrophoresis of fraction No 25 was performed and the gel was stained as described before^69^. The resolved proteins were electroblotted on a PVDF membrane, stained using Coomassie Blue, visualised using the Chemi Doc MP ImagingSystem (Bio-Rad, Hercules, CA, USA), and cut out from the membrane for identification.

### GmSPID protein purification

Eight hundred microliters of cell-free hemolymph from larvae immunised with *E. coli* and *M. luteus* were mixed with 800 µl of solvent A (0.1% v/v trifluoroacetic acid, TFA), 400 µl of solvent B (0.07% TFA containing 80% acetonitrile, both v/v), and 13 µl of TFA. The mixture was shaken for several minutes and then centrifuged at 20,000 × g for 15 min at room temperature. The pellet of insoluble denatured proteins was discarded, while the clear supernatant was subjected to reversed-phase high pressure chromatography (RP-HPLC) using an UltiMate 3000 HPLC apparatus (Thermo, Waltham, MA, USA) and a Discovery Bio Wide Pore C18 4.6 mm × 250 mm column (Sigma-Aldrich, St. Louis, MO, USA). The chromatography was carried out at 40 °C using a biphasic gradient of solvents A and B mentioned above (20–67% of solvent B in 45 min). The flow rate was 1 ml/min, while the spectrophotometric detection was performed at 220 and 280 nm. The fraction eluting at ca. 15.0–17.3 min was collected and subjected to re-chromatography using the same column as above and a linear gradient from 27 to 33% of solvent B within 30 min developed at 50 °C. The fraction eluting at 24.6 min was collected and subjected once again to chromatography using a Discovery Bio Wide Pore C8 4.6 mm × 250 mm column (Sigma-Aldrich, St. Louis, MO, USA) and a linear gradient from 21 to 31% of solvent B within 20 min developed at 45 °C. The peak eluting at 15.0 min, containing pure GmSPID protein, was collected, freeze dried, and dissolved in water. The homogeneity and identity of the purified protein were confirmed by SDS-PAGE electrophoresis and N-terminal amino acid sequencing performed using an automatic protein sequencer (PPSQ-31 A, Shimadzu, Kyoto, Japan). The concentration of the protein in the solution was determined with the use of a bicinchoninic acid assay (BCA, Sigma-Aldrich, St. Louis, MO, USA) calibrated using bovine serum albumin.

### Determination of the relative expression of the GmSPID gene in the fat bodies of *G. mellonella* larvae

Total RNA from the *G. mellonella* fat body was isolated with the use of a Gen Elute Mammalian Total RNA Extraction Kit (Sigma) according to producer’s protocol and its quality was assessed by estimation of the 260/280 ratio. One microgram of RNA was used for reverse transcription performed with the use of a High-Capacity cDNA Reverse Transcription Kit (Life Technology) according to supplier’s protocol using random hexamer primers. Quantitative PCR was performed using the Step One Plus System (Applied Biosystems) in the following conditions: 95 °C 10 min, 44 × (95 °C, 15 s—denaturation; 60 °C, 1 min—annealing and extension). The primers for ribosomal protein gene S7e, serving as a reference gene, were published before^70^. The primers for GmSPID were as follows: forward 5ʹ-GTGTCATCTGTGGACCGTCAGT-3ʹ, reverse 5ʹ-TTAAGGCCAATATGCGAACGA-3ʹ. The relative gene expression was calculated taking into account the efficiency of reaction, which was ≥ 95% ^71^. In each of the three experiments, fat bodies from 5 larvae in every group were pooled and used for RNA extraction. More details can be found elsewhere^15,20^.

### Proteolytic assays

To avoid enzyme excess, the minimum amount of each enzyme giving the maximum product was used for proteolytical analysis. GmSPID at the indicated concentrations was mixed with 1 pmol of thermolysin (from *Bacillus thermoproteolyticus rocco*, Sigma-Aldrich) in acetate buffer (10 mM sodium acetate, 5 mM calcium acetate, pH 7.5), or with 6 pmoles of trypsin (from bovine pancrease, Sigma-Aldrich) in reaction buffer (10 mM Tris HCl, 1 mM HCl, pH 8.0), or with 0.02 U of elastase (from porcine pancreas, Sigma; minimum 4 U/mg stock solution ) in 10 mM Tris-HCl pH 8.0 buffer in a total volume of 5 µl. In the control group, an equal volume of water was added instead of GmSPID. The samples were pre-incubated for 15 min at room temperature. Then, an equal volume of an azocasein (Sigma-Aldrich) solution (5 mg/ml in water) was added, and the samples were incubated for 1 h at 30 °C. The reaction was stopped by adding 10 µl of 5% trichloroacetic acid (TCA, Sigma-Aldrich), followed by 10-min incubation at room temperature. After the incubation, the samples were centrifuged at 14,000 × g for 5 min to precipitate undigested casein. 18 µl of the supernatant containing azopeptides was mixed with 9 µl of a 0.5 M NaOH solution. The absorbance of the samples was measured at 450 nm against a blank sample on clear half-area flat-bottom 96 well plates (Corning) with the use of a Benchmark Plus microplate reader (BioRad). EDTA, soybean trypsin inhibitor (STI), and phenylmethylsulfonyl fluoride (PMSF) were used at the indicated concentrations as positive inhibition controls for thermolysin, trypsin, and elastase activity assays, respectively (all reagents from Sigma-Aldrich). Each assay was performed in at least three replicates.

### Antimicrobial activity assay

The assay was performed as described before^15^. Shortly, twenty microliters of exponentially grown bacteria (Table 1) were diluted in LB to OD_600_ of 0.02 and mixed with 4 µl the GmSPID peptide or water as a control and quickly divided into two parts of 10 µl each. One pair (bacteria with GmSPID and with water) of samples was incubated for 60 min with shaking at a temperature depending on the microorganism (Table 1) and then plated (time 60), while the second pair was plated immediately (time 0). For plating, serial dilutions from 10^−2^ to 10^−4^ in LB were prepared, and two last dilutions were mixed with LB containing soft agar (0.7%w/v) cooled down to 40 °C and poured on Petri plates. The plates were incubated at 30–37 °C (depending on the microorganism) until colonies were clearly visible (overnight). The results are presented as a percentage of CFU that appeared after plating the GmSPID-containing samples in relation to the respective CFU obtained from samples without the peptide.

For testing the activity against *C. albicans* (see Table 1), logarithmically growing fungi diluted to OD_600_ of 0.0025 were mixed with GmSPID and directly divided into two portions. One portion was incubated at 37 °C for 60 min, while the other part was plated directly (time 0). For plating, the mixtures were diluted 15 and 150 times, and each dilution was mixed with YPD medium containing soft agar (0.7%) and plated on Petri dishes. The number of CFU after mixing the fungi with water (instead of GmSPID) was taken as 100%. More details are described in^15^.

### Bacterial membrane permeabilisation assay

GmSPID was pre-incubated for 15 min at 37 °C in 20 mM phosphate buffer pH 6.8. Next, 2 µl of a suspension of mild-logarithmic phase *E. coli* JM83 cells (5 × 10^5^ CFU) carrying plasmid pCH110 encoding ß-galactosidase (Pharmacia-Amersham) in the same buffer was added to 23 µl of the GmSPID solution. After 45-min incubation at 37 °C, 20 mM HEPES buffer pH 7.5 containing 150 mM NaCl (220 µl) and a 50 mM p-nitrophenyl-β-d-galactopyranoside solution (5 µl, substrate for leaking ß-galactosidase from permeabilised cells) were added to the mixture. The samples were incubated for 90 min at 37 °C, and the absorbance at 405 nm was measured. Live bacteria incubated with water in the growth medium were used as a negative control (0% perforation), and dead bacteria killed by 5 µM of synthetic cecropin B (Sigma-Aldrich) served as a positive control (100% perforation)^15,72^.

### Atomic force microscopy

*P. entomophila* was incubated with GmSPID (or water as a control) as described above for the antibacterial assay but in a total volume of 300 µl. Then, an equal volume of 20 mM phosphate buffer pH 6.8 was added, and the samples were centrifuged at 7000 × g for 10 min. The pellets were washed twice with the same buffer as above and twice with non-pyrogenic water. The bacteria were suspended in 10 µl of non-pyrogenic water, applied onto freshly cleaved mica discs, and left for overnight drying at room temperature before imaging. The surface of *P. entomophila* was imaged using NanoScope V AFM (Veeco, USA) in the Analytical Laboratory, Faculty of Chemistry, Maria Curie-Sklodowska University in Lublin, Poland. The measurements were performed as described in^15^.

### SEM and TEM microscopy

For SEM, the antimicrobial assay was performed as described above in the section (Antibacterial activity assay) but in the volume of 500 µl for the control and the GmSPID-treated samples. For microscopy, the samples were prepared according to the protocol published elsewhere^73^. First, the bacteria were fixed with a solution of 4%glutaraldehyde in 0.1 M cacodylate buffer (pH 7.2) for 2 h at 40 °C. Then, the samples were rinsed three times (each for 15 min) in 0.1 M cacodylate buffer. Subsequently, the cell pellets were post-fixed in a 1% (v/v) osmium tetroxide solution in cacodylate buffer (pH 7.2) for 1 h at 40 °C. After dehydration in a graded series of ethanol (30%, 50%, 70%, 90%, 100%) for 15 min in each, the samples were dried with CO_2_ in a critical point drying chamber and coated with gold in an Emitech K550X Sputter Coater. Afterwards, the samples were imaged using a TESCAN vega 3 LMU microscope (Czech Republic) in the secondary electron mode.

For TEM, the assay was performed in the volume of 800 µl. Then, the bacteria were centrifuged at 8000 × g for 10 min. The pellets were then washed with 0.1 M sodium phosphate buffer, pH 7.2, two times. Next, the supernatants were removed, replaced with 3% glutaraldehyde in 0.1 M phosphate buffer, and incubated at 40 °C for 2 h. After fixation, the samples were rinsed 3 times (each 20 min) in 0.1 M sodium phosphate buffer. The samples were then treated with a 1% solution of osmium tetroxide in sodium phosphate buffer (0.1 M; pH 7.2) for 1 h. After washing with PBS (three times for 20 min each), dehydration with increasing concentrations of ethanol was performed. Then, the samples were embedded in LR White resin (MERC, Germany) and polymerised at 55 °C. Ultrathin Sect. (60 nm) were cut with a diamond knife on 300 copper mesh grids with the use of a Leica EM UC7 ultramicrotome. After double staining with lead citrate and uranyl acetate, the samples were examined with a JEOL JEM 1400 Flash electron microscope^73^.

### Statistical methods

Statistical analysis was performed using Sigma Plot 12.5 (Systat Software Inc., USA). Normality of data was assessed with the use of the Shapiro–Wilk test. Significant differences were established at *p* < 0.05. For comparison of more than two groups, One-way ANOVA and post-hoc tests were used. Significant differences were established at *p* < 0.05. Values marked with the same letters do not different significantly. The atomic force microscopy results were analysed using the Mann–Whitney U test. Significant differences are indicated with *p < 0.05, **p < 0.01, and***p < 0.001.

### Other methods

Mass spectrometry measurements were performed by the service at the Proteomics and Mass Spectrometry Core Facility of the Małopolska Centre of Biotechnology, Jagiellonian University, Kraków, Poland.

## Electronic supplementary material

Below is the link to the electronic supplementary material.

Supplementary Material 1

## Data Availability

The data that support the findings presented in this article are openly available in Zenodo at https://zenodo.org, record 14843494.
