# Supplementary material for: Serine protease inhibitor dipetalogastin-like from Galleria mellonella is involved in insect immunity
Source: Sci Rep. 2025 Jul 6;15:24094. doi: 10.1038/s41598-025-08159-z (PMC12230126; doi:10.1038/s41598-025-08159-z)
Supplement: Supplementary file 1 — Supplementary Material 1 [file 41598_2025_8159_MOESM1_ESM.pdf]

Serine protease inhibitor dipetalogastin-like from *Galleria mellonella* is involved in insect immunity

Jakub Kordaczuk[1], Michał Sułek[1a], Paweł Mak[2], Bożena Pawlikowska-Pawlęga[3], Iwona Wojda[1*]

[1] Maria Curie-Sklodowska University, Institute of Biological Sciences, Department of Immunobiology, Lublin, Akademicka 19 Str., 20-033 Lublin, Poland

[2] Jagiellonian University, Faculty of Biochemistry, Biophysics and Biotechnology, Department of Analytical Biochemistry, Gronostajowa 7 Str., 30-387 Kraków, Poland

[3] Maria Curie-Sklodowska University, Institute of Biological Sciences, Department of Functional Anatomy and Cytobiology, Lublin, Poland

[a] present address Maria Curie-Sklodowska University, Institute of Biological Sciences, Department of Virology and Immunology, Lublin, Akademicka 19 Str., 20-033 Lublin, Poland

Supplementary materials. Table and Figs. S1, S2, S3, S4, S5 and S6

Supplementary Table

Comparison of GmSPID protein (predicted full protein sequence is provided) from *G. mellonella* to other proteins with the use of NCBI database (BLAST).

Percent of amino acid sequence identity to *G. mellonella* IPSD	Query Cover	Protein	Predicted length (aa)/predicted molecular mass (kDa)	Organism
**100 %**	**100 %**	**XP_026756133**<br>**serine protease inhibitor dipetalogastin-like**<br>**predicted full aminoacid sequence:**<br><br>1- *mykygillla vylstasa*lp pcvctrdyrp<br>vcgsngetyp nkcmlncaqa tnrqitlkqs<br>gpcdspaqpi cvctfeykpv cgsdgktypn<br>rcslnceasv rlahegncae pvkvaqlprc<br>tctkekqpvc gsdgatysnd cmlncatqfn<br>pslrishfgp cnsevkvvee ssdntvapct<br>ctrelkpvcg sdgqtytsec imrcrnkyat<br>varegpcel- 219	**219 /23.65**	***Galleria mellonella***
77.98 %	99 %	XP_059048673.1<br>serine protease inhibitor dipetalogastin-like	217/23.376	*Achroia grisella*
53.45 %	99 %	XP_047032450.1<br>thrombin inhibitor rhodniin-like	178/19.033	*Helicoverpa zea*
52.87 %	99 %	XP_063895205.1<br>thrombin inhibitor rhodniin-like	178/19.038	*Helicoverpa armigera*

52.8 %	91 %	XP_053610273.1<br>serine protease inhibitor dipetalogastin-like	278/29.073	*Plodia interpunctella*
52.7 %	99 %	XP_046969648.1<br>serine protease inhibitor dipetalogastin-like	216/23.66	*Vanessa cardui*
52.68 %	99 %	XP_045451539.1<br>serine protease inhibitor dipetalogastin	244/24.176	*Melitaea cinxia*
52.66 %	97 %	PZC81105.1<br>hypothetical protein B5X24_HaOG213445<br>(contains Kazal domains)	180/19.46	*Helicoverpa armigera*
52.47 %	99 %	CAH2093169.1<br>unnamed protein product<br>(contains Kazal domains)	224/23.96	*Euphydryas editha*
52.25 %	97 %	XP_030025305.1<br>serine protease inhibitor dipetalogastin	285/30.7	*Manduca sexta*
52.21 %	99 %	XP_063386583.1<br>serine protease inhibitor dipetalogastin-like	221/23.653	*Cydia fagiglandana*

Supplementary Figures

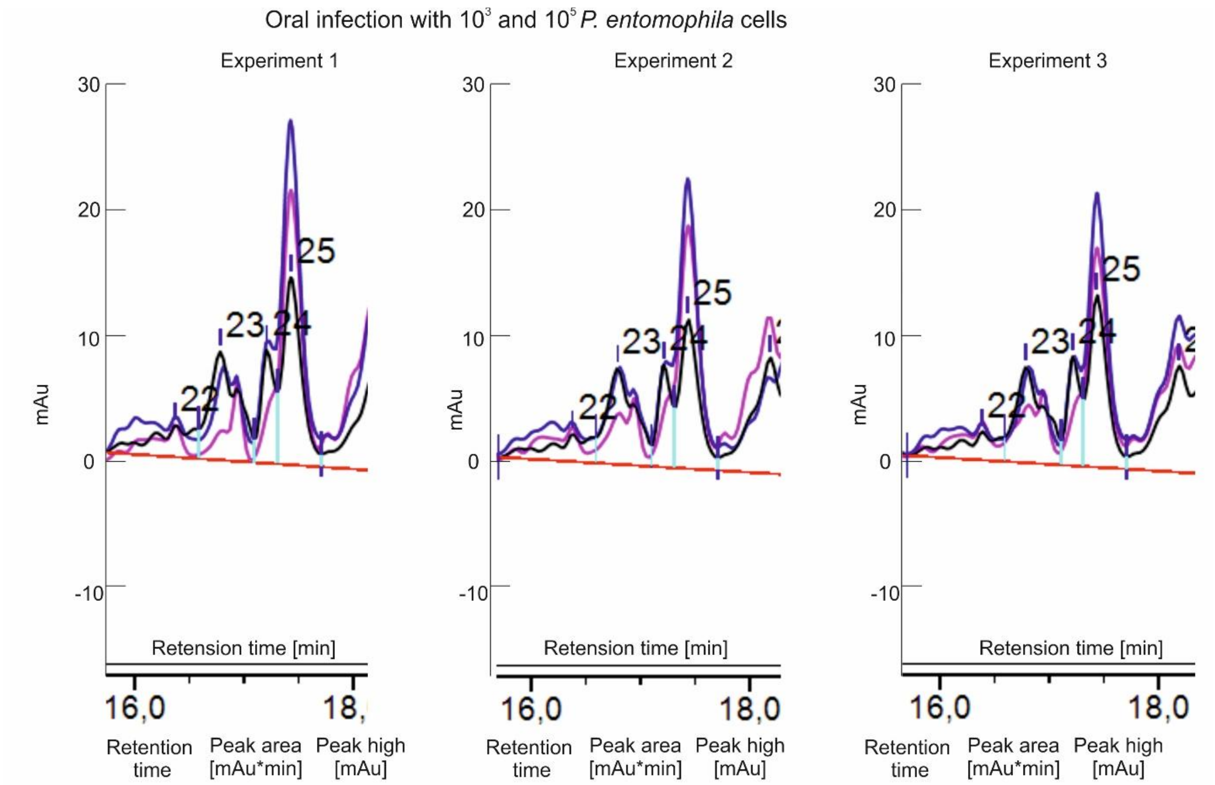

	Experiment 1: Retention time	Experiment 1: Peak area [mAu*min]	Experiment 1: Peak high [mAu]	Experiment 2: Retention time	Experiment 2: Peak area [mAu*min]	Experiment 2: Peak high [mAu]	Experiment 3: Retention time	Experiment 3: Peak area [mAu*min]	Experiment 3: Peak high [mAu]
PBS	17,427	4,684	22,52	17,433	4,2291	19,66	17,433	3,782	17,6
Pe 10^3	17,423	5,6065	27,52	17,43	4,7066	23,04	17,433	4,4676	21,77
Pe 10^5	17,427	2,9546	14,93	17,433	2,3909	11,92	17,433	2,6973	13,73

Supplementary Figure S1. Superimposed part of chromatograms presenting fraction No 25 from the RP-HPLC separation of low molecular weight polypeptides from the hemolymph of larvae receiving orally PBS (pink lines), 103 cells (navy blue lines), and 105 cells (black lines) of P. entomophila. Entire chromatograms were published in [41]

Intrahemocelic injection with 10 and 50 cells of *P. entomophila*

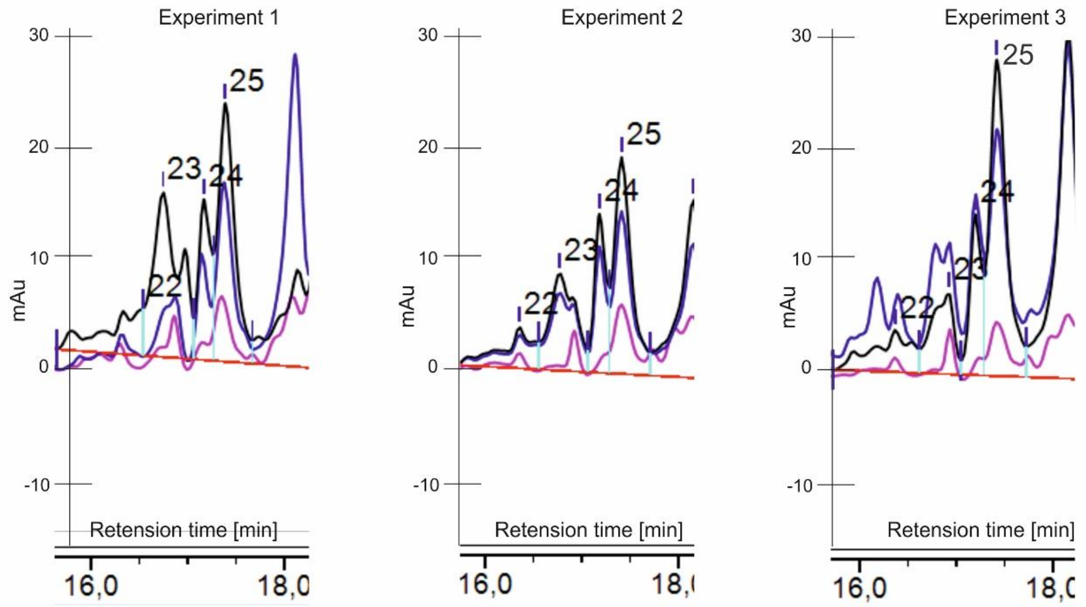

	Experiment 1 Retention time	Experiment 1 Peak area [mAu*min]	Experiment 1 Peak high [mAu]	Experiment 2 Retention time	Experiment 2 Peak area [mAu*min]	Experiment 2 Peak high [mAu]	Experiment 3 Retention time	Experiment 3 Peak area [mAu*min]	Experiment 3 Peak high [mAu]
PBS	17,347	1,8033	7,59	17,403	1,5755	6,89	17,417	1,3828	5,73
Pe 10	17,373	3,8117	17,42	17,407	3,2877	14,71	17,42	4,4695	20,93
Pe 50	17,387	4,6355	23,27	17,41	4,041	19,51	17,417	5,8465	28,48

Supplementary Figure S2. Superimposed part of chromatograms presenting fraction No 25 from the RP-HPLC separation of low molecular weight polypeptides from the hemolymph of larvae injected with PBS (pink lines), 10 cells (navy blue lines), and 50 cells (black lines) of P. entomophila. Entire chromatograms were published in [41]

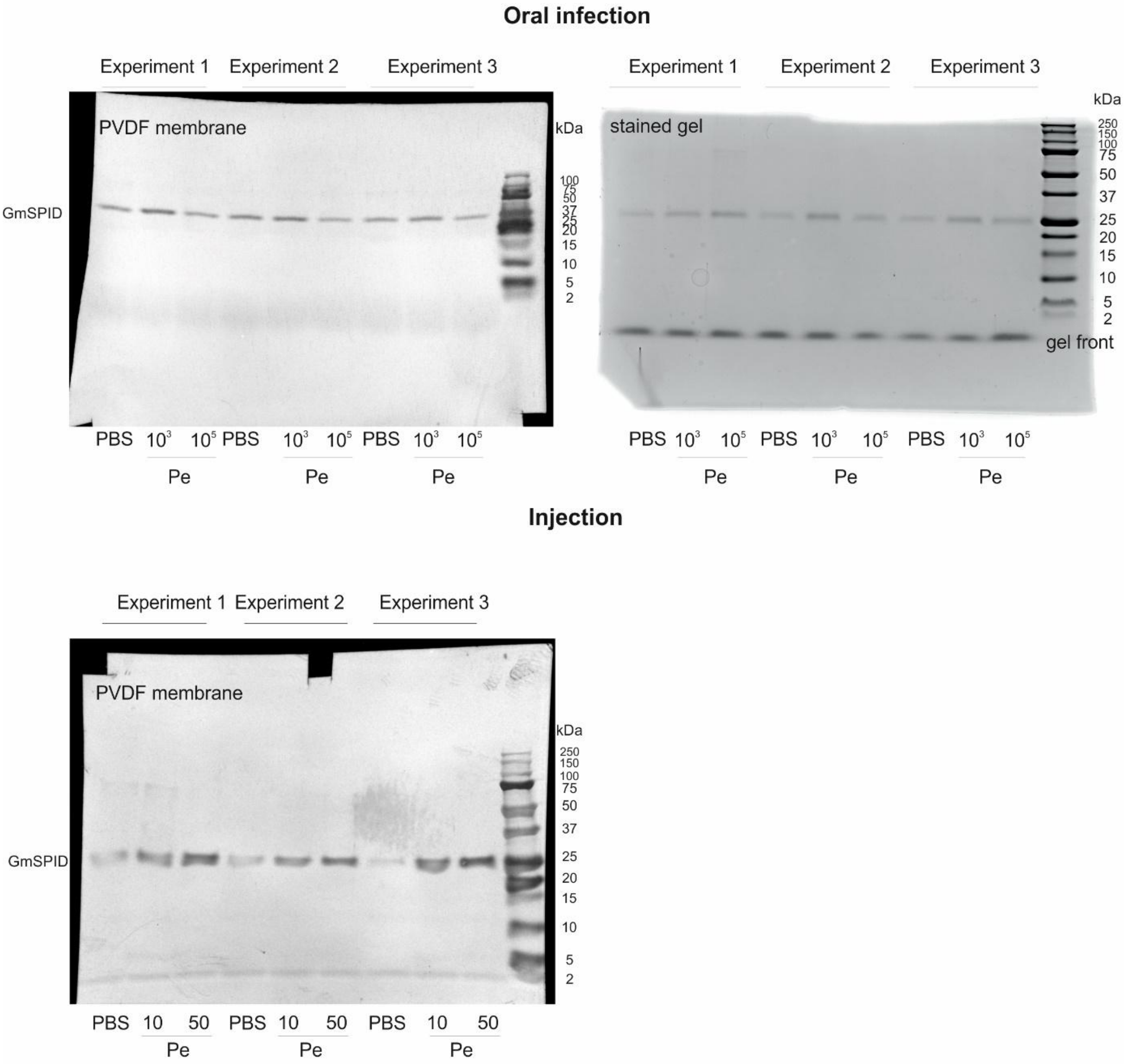

Supplementary Figure S3

Uncropped and uncut images of PVDF membranes presented in Fig. 1 (left).
Electrophoresis of fraction No25 (oral administration) was repeated to obtain
better separation of molecular mass standards and the image of stained gel is presented (right).

A. IDENTIFICATION OF P25 PROTEIN BY MASS SPECTROMETRY

Accession	Description	Score	Coverage	# Proteins	Unique Peptic	# Peptides	# PSMs	# AAs	MW [kDa]	calc. pI
**XP_026756133.1**	**serine protease inhibitor dipetalogas**	**4933,74**	**76,71**	**1**	**13**	**13**	**137**	**219**	**23,8**	**7,44**

Sequence	Modifications	IonScore	# PSMs	Charge	m/z [Da]
LAHEGNcAEPVK	C7(Carbamidor	53	14	2	662,81805
ISHFGPcNSEVK	C7(Carbamidor	58	7	2	687,82703
LAHEGNcAEPVKVAQLPR	C7(Carbamidor	41	1	3	663,68286
ELKPVcGSDGQTYTSEcImR	C6(Carbamidor	95	26	2	1174,02039
EKQPVcGSDGATYSNDcmLNcATQFNPSLR	C6(Carbamidor	99	13	3	1146,15845
EKQPVcGSDGATYSNDcMLNcATQFNPSLR	C6(Carbamidor	102	3	3	1140,82849
ELKPVcGSDGQTYTSEcIMR	C6(Carbamidor	82	4	2	1166,02380
DYRPVcGSNGETYPNK	C6(Carbamidor	60	5	3	619,61163
QSGPcDSPAQPIcVcTFEYKPVcGSDGK	C5(Carbamidor	53	2	3	1048,78882
QPVcGSDGATYSNDcmLNcATQFNPSLR	C4(Carbamidor	89	8	3	1060,44714
QPVcGSDGATYSNDcMLNcATQFNPSLR	C4(Carbamidor	72	3	3	1055,11633
VVEESSDNTVAPcTcTR	C13(Carbamidc	101	15	2	962,92157
cmLNcAQATNR	C1(Carbamidor	73	12	2	677,78650
cSLNcEASVR	C1(Carbamidor	83	9	2	598,26477
cMLNcAQATNR	C1(Carbamidor	66	2	2	669,78925
YATVAR		43	1	2	340,69009
VAOLPR		44	12	2	342,21356

serine protease inhibitor dipetalogastin-like [Galleria mellonella]

☐ Annotate PTMs reported in Uniprot
☐ Show only PTMs
☐ Include PSMs that are filtered Out

Coverage: 76.71%

Found Modifications:

C Carbamidomethyl (C)
O Oxidation (M)

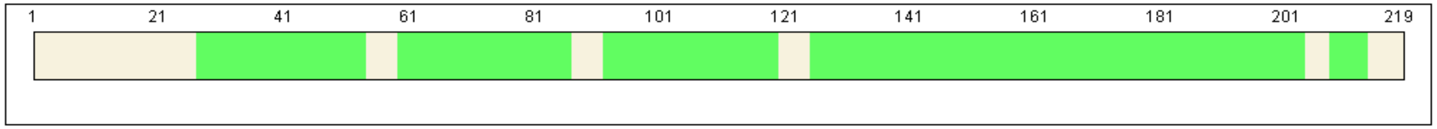

Sequence | Modification List

```
     1          11         21         31         41         51         61         71         81         91         101
                                       C                     CO   C                     C          C  C       C           C    C               C
  1  MYKYGILLLA VYLSTASALP PCVCTRDYRP VCGSNGETYP NKCMLNCAQA TNRQITLKQS GPCDSPAQPI CVCTFEYKPV CGSDGKTYPN RCSLNCEASV RLAHEGNCAE
                                   C            CO   C                C                C  C          C           C   O
111  PVKVAQLPRC TCTKEKQPVC GSDGATYSND CMLNCATQFN PSLRISHFGP CNSEVKVVEE SSDNTVAPCT CTRELKPVCG SDGQTYTSEC IMRCRNKYAT VAREGPCEL
```

B. N-TERMINAL SEQUENCE OF P25 PROTEIN BY EDMAN DEGRADATION:

LPPCVCTRDYR

Aminoacids 1 -18 - signal seguence

Supplementary Figure S4. Identification of P25 protein by Mass spectometry (A) and N-terminal sequencing (B).

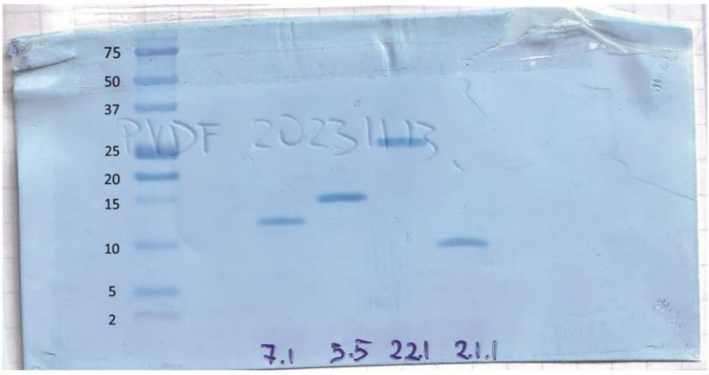

Supplementary Fig. S5. Uncut and uncropped stained membrane presenting the purified GmSPID

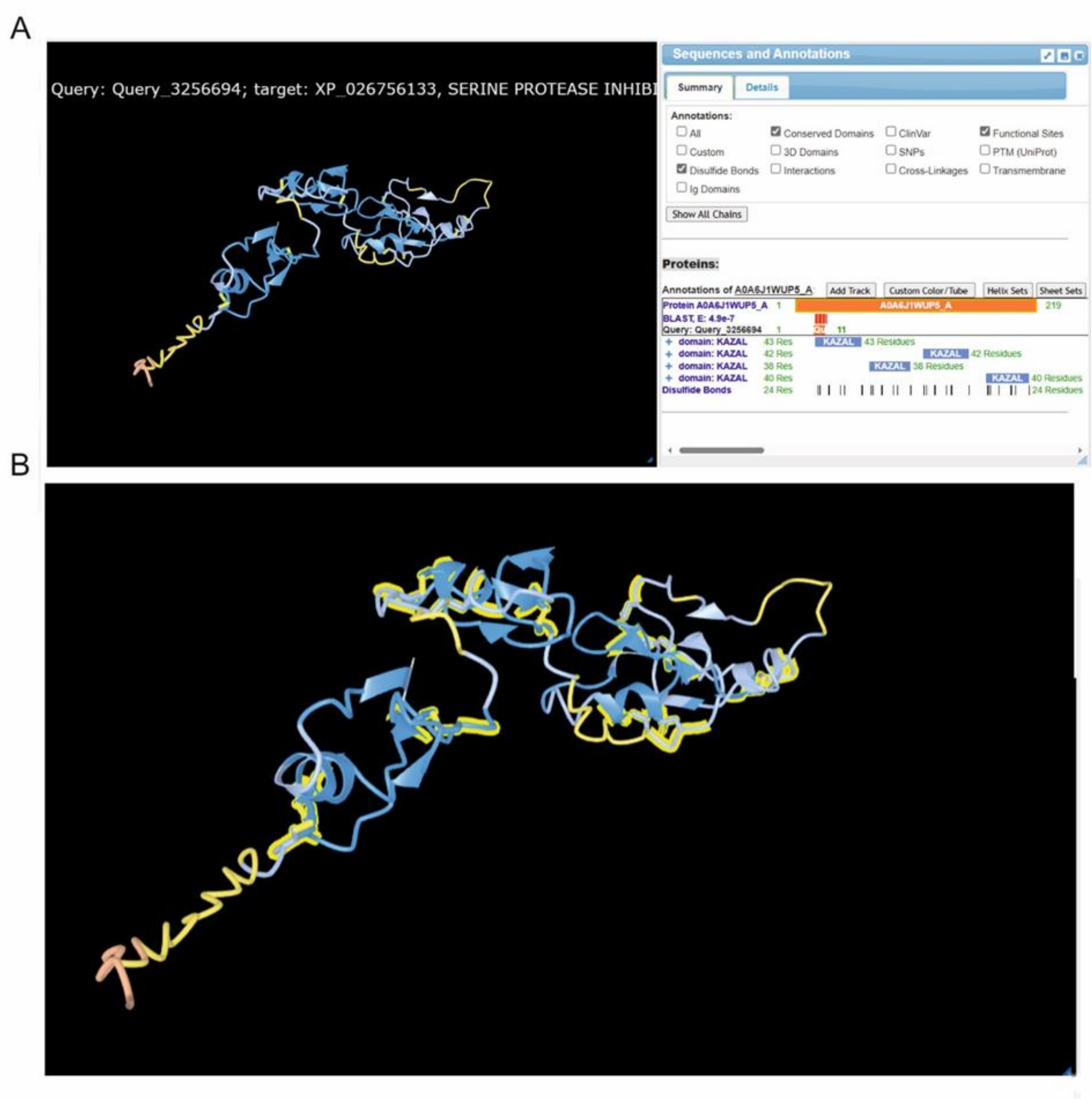

Supplementary Figure S6. A putative structure of GmSPID generated by NCBI BLAST (www.ncbi.nlm.nih.gov)- left hand site with localisation of Kazal domains (right hand side). B- localisation of disulfide bridges within GmSPID structure is indicated by surrounding yellow lines.
